# Treatment Related Impairments in Arm and Shoulder in Patients with Breast Cancer: A Systematic Review

**DOI:** 10.1371/journal.pone.0096748

**Published:** 2014-05-09

**Authors:** Janine T. Hidding, Carien H. G. Beurskens, Philip J. van der Wees, Hanneke W. M. van Laarhoven, Maria W. G. Nijhuis-van der Sanden

**Affiliations:** 1 Radboud university medical center, Department of Orthopedics, Section of Physical Therapy, Nijmegen, The Netherlands; 2 Radboud university medical center, Scientific Institute for Quality of Healthcare, Nijmegen, The Netherlands; 3 Academic Medical Center, Department of Medical Oncology, University of Amsterdam, Amsterdam, The Netherlands; Supportive care, Early DIagnosis and Advanced disease (SEDA) research group, United Kingdom

## Abstract

**Background:**

Breast cancer is the most common type of cancer in women in the developed world. As a result of breast cancer treatment, many patients suffer from serious complaints in their arm and shoulder, leading to limitations in activities of daily living and participation. In this systematic literature review we present an overview of the adverse effects of the integrated breast cancer treatment related to impairment in functions and structures in the upper extremity and upper body and limitations in daily activities. Patients at highest risk were defined.

**Methods and Findings:**

We conducted a systematic literature search using the databases of PubMed, Embase, CINAHL and Cochrane from 2000 to October 2012, according to the PRISMA guidelines. Included were studies with patients with stage I–III breast cancer, treated with surgery and additional treatments (radiotherapy, chemotherapy and hormonal therapy). The following health outcomes were extracted: reduced joint mobility, reduced muscle strength, pain, lymphedema and limitations in daily activities. Outcomes were divided in within the first 12 months and >12 months post-operatively. Patients treated with ALND are at the highest risk of developing impairments of the arm and shoulder. Reduced ROM and muscle strength, pain, lymphedema and decreased degree of activities in daily living were reported most frequently in relation to ALND. Lumpectomy was related to a decline in the level of activities of daily living. Radiotherapy and hormonal therapy were the main risk factors for pain.

**Conclusions:**

Patients treated with ALND require special attention to detect and consequently address impairments in the arm and shoulder. Patients with pain should be monitored carefully, because pain limits the degree of daily activities. Future research has to describe a complete overview of the medical treatment and analyze outcome in relation to the treatment. Utilization of uniform validated measurement instruments has to be encouraged.

## Introduction

Breast cancer is the most common type of cancer in women in the developed world. Due to new treatment modalities, breast cancer survival has improved over time. However, as a result of breast cancer treatment, many patients suffer from adverse effects and have serious complaints in their arm and shoulder e.g. decreased joint mobility, muscle strength, pain and lymphedema, leading to limitations in activities of daily living and participation in work, sports and leisure activities. [1]–[3] In a prospective Australian study, 62% of the population still suffered from at least one impairment as a complication of breast cancer treatment and 27% suffered from two to four impairments after six years. [4] Reported variability in onset and severity of upper limb symptoms of patients with breast cancer reported in studies is large [5] and a systematic overview of risk factors related to medical treatment is lacking. This information is of direct clinical relevance, as early physical therapy intervention for these complaints as well as surveillance of patients at risk for developing impairments in daily activities reduces the need for intensive rehabilitation and the associated costs. [6] Based on the misconception that disabilities such as decreased range of motion, pain and lymphedema will resolve over time without intervention, combined with denial of the possible benefits of physical therapy interventions, this has led to the inadequate monitoring of disabilities. [7] To the best of our knowledge, this is the first systematic review with an evidence synthesis on the physical adverse effects of all components of breast cancer treatment, analyzed for each treatment modality, on impairments in the arm and shoulder, leading to limitations in activities that potentially warrant treatment. If the clinician is aware of the risk of adverse effects of the treatment, clinical reasoning regarding surveillance and the early detection of impairments in patients at risk can be applied in a systematic way.

In this article, we present a systematic literature review of the adverse effects of breast cancer treatment in terms of development of constraints in the arm and shoulder in patients with stage I–III breast cancer who underwent curative treatment. We describe the adverse effects for treatment-induced disorders of the musculoskeletal system - classified by International Classification of Functioning, Disability and Health (ICF) domains [8] - and assess the influence of pre-existing comorbidity. More specifically, the following key question is answered in this systematic review: which adverse effects related to breast cancer treatment predict persistent impairments in function and structures of the upper extremities/thorax, e.g. reduced joint mobility, reduced muscle strength, pain, lymphedema and limitations in daily activities?

## Methods

### Study selection criteria

#### Search strategy

We conducted a systematic literature search using the databases of PubMed, Embase, CINAHL and Cochrane. Published studies in English, French and German language were eligible for inclusion. We started with the inclusion of eligible meta-analyses and systematic reviews, and then considered the inclusion of prognostic cohort studies, case-control studies and cross-sectional studies that were not included in published systematic reviews. To minimize bias, only studies with at least 100 patients were included. Studies which had already been included in systematic reviews or meta-analyses were not analyzed separately. To allow for an adequate follow-up and description of late adverse effects, only studies with a follow-up period of at least 3 months were included. When more publications of the same study were published, data were extracted from the most recent publication. As we were merely interested in adverse effects in relation to current medical practice, studies published from January 2000 to October 2012 were included. The search strings are listed in table 1.

**Table 1:** Search string adverse effects.

Pubmed	(((((("Breast Neoplasms" [Mesh] OR "Breast Neoplasms" OR "breast cancer")) AND (surgery))) AND (((((radiotherapy)) OR (((("Breast Neoplasms/drug therapy" [mesh])) OR ("Antineoplastic Agents" [Mesh])) OR ("chemotherapy" [All Fields]))) OR ("Antineoplastic Agents" [Pharmacological Action])) OR (hormonal therapy)))) AND (((((((((activities)) OR ("Activities of Daily Living" [Mesh]))) OR (range of motion)) OR (("Muscle Strength" [Mesh]) OR "Range of Motion, Articular" [Mesh])) OR (muscle strength)) OR (Lymphedema)) OR (pain)) AND (dutch [la] OR english [la] OR german [la] OR french [la]) AND ("2000/01/01" [PDAT] : "3000/12/31" [PDAT])
Cinahl	TI breast cancer AND ((AB "Range of Motion" ) OR (AB "Muscle Strength”) OR (AB Lymph*) OR (AB “Activities of Daily Living” ) OR (AB pain)) Limiters: Published Date from: 20000101–20121231 Language English
Embase	breast cancer.ti. AND ((activities of daily living.ab.) OR (range of motion.ab.) OR (muscle strength.ab.) OR (muscle strength.ab.) OR (Lymphedema.ab.) OR (pain.ab.)) Limit to (english language and yr = “2000– 2012”)
Cochrane	Topic ‘breast cancer’ AND ‘adverse effects’

#### Patients

Studies on patients with curatively treated breast cancer (Stage I–III) were included.

#### Intervention

Included medical interventions were: surgery (mastectomy, lumpectomy, axillary lymph node dissection [ALND], sentinel node biopsy [SNB], and breast reconstruction) and additional treatments (radiotherapy, chemotherapy and hormonal therapy).

#### Outcomes

The following health outcomes were extracted: impairment in functions and structures in the upper extremity and upper body (reduced joint mobility, reduced muscle strength, pain, and lymphedema), and limitations in daily activities of the upper extremity. Outcomes had to be measured with instruments for which validation studies were published, or for which the authors described validation before initiation of the study.

Description of adverse effects of the medical treatment was divided into effects within the first 12 months and late effects (>12 months). When outcome measures of severe cases were presented as well, these were presented between brackets in table 2.

**Table 2:** Outcome of the studies regarding breast cancer treatment and adverse effects.

Author/year of publication	Design	Disease stage/treatment/number of pts included	Number of studies/Dates of inclusion/FU in months (% FU if mentioned)	Measurement instruments in outcome	Main findings
**Hickey et al. 2013**	SR	Concurrent RT + CT vs. sequential n = 107/107/RT then CT vs. CT then RT, n = 117/119 for LE; n = 42/43 for brachial neuropathy	3 studies: RCT; 3 survival, 2 toxicity/Up till Dec. 2011; 60/135months (FU 74%)	CTCAE/LENT-SOMA	Late toxicity 29% ; **Concurrent vs. sequential RT after CT:** Grade III/IV, in favour of sequencing: atrophy OR = 2.09 (CI = 0.92–4.75); fibrosis OR = 13.77 (CI = 0.77–247.54);LE OR = 2.02 (CI = 0.18 to 22.61). **RT before CT vs. CT before RT:** In favour of RT first: LE OR = 2.11 (CI = 0.67–7.21) ; Brachial neuropathy OR = 3.14 (CI = 0.12–79.39)
**Moja et al. 2012**	SR	Stage I-III/HER2 pos. BC/Trastuzumab + CT vs. CT alone(Anthracyclines, Taxanes,Vinorelbine, other CT); CHF n = 5471/4810; LVEF n = 4147/3792	8 studies: RCT 8/1996-Feb. 2010/% FU missing/≥ 24 months	Cardiac toxicity (CHF, LVEF), other toxicities	**Trastuzumab vs. no trastuzumab:** CHF↑, cardiac toxicity↑, LVEF↓; CHF: trastuzumab administration >6 months OR = 5.11; Cardiac toxicity: trastuzumab before CT OR = 8.42; CT before trastuzumab OR = 11.05; Concurrent CT/trastuzumab OR = 3.90 (overall >6 months OR = 5.12); LVEF ↓ OR = 1.83; < 6 months OR = 0.89; >6 months OR = 2.14. **Trastuzumab before CT:** OR = 1.16. **CT before trastuzumab:** OR = 2.90, Concurrent **CT/trastuzumab:** OR = 1.48
**Zhou et al. 2011**	SR	Stage I–IV/Zoledronic acid/ZOL vs. no ZOL n = 2684/2712/Delayed ZOL vs. upfront ZOL n = 119/284	4 studies: RCT 4/Up till May 2011 (Art 1. CT [mostly anthracycline] +/− HT; Art 2. Gosselerin + tamoxifen or anastrozole; Art 3/4 adjuvant treatment not specified/% FU missing/12–60 months	Not described	**ZOL vs. no ZOL:** ↑ arthralgia (4 studies); ↑ bone pain (2 studies); arthralgia RR = 1.16; bone pain RR = 1.26; muscle pain no differences between groups; complications 0.2–0.8% per item. **Delayed vs. upfront ZOL:** No differences between groups for bone pain/arthralgia; arthralgia RR = 1.28. **Anastrozole alone vs.. tamoxifen alone:** arthralgia 25% vs. 12% ; bone pain (28% vs. 21%) (art 2). **Anastrozole + ZOL vs. tamoxifen + ZOL:** bone pain 35% vs. 25%; arthralgia 24% vs. 18% (art 2)
**Levangie et al. 2009**	SR	ALND/SNB/RT/Breast cancer vs. non breast cancer n = 1501/ALND vs. SNB vs. none/n = 2353/996/59	36 studies: CS 7; CCT 11; prospective 10; retrospective 1; CSS 2; RCT 5/1980–2008/% FU missing/12–126 months	ROM, muscle strength/grip strength/upper body functions	**ALND vs. SNB or non-affected side:** ROM↓ flexion, abduction and abduction/external rotation; OR = 1.02/2.65/9.0*. Muscle strength ↓ grip strength, resistance abduction; OR = 8.82. Pain OR = 3.54 (1.88–6.66). Upper arm activities ↑ limitations compared to non-breast cancer; ↓: ALND OR = 3.18/9.23*. **RT vs. no RT:** OR = 1.32/2.64/4.67*
**Liu et al. 2009**	SR	SNB vs. SNB + ALND vs. ALND/RT/n = 7135 vs. 1225 vs. 1445.	17 studies: RCT 5, CCT 12: prospective 9, retrospective 3/SNB vs. SNB + ALND vs. ALND/1993–2008/% FU missing/6–72 months	ROM, Hand-held dynamometer, MPQ, VAS, tape measurement, MASS	**SNB:** *6 months:* LE 3–10%. *12 months:* ROM↓ 6–31%; RT OR = 2.6; muscle strength ↓ 17–19%; pain 8–36%; LE 6–14%. *24 months:* Pain 8–21%; upper arm activities↓: RT axilla OR = 2.6. *36 months:*ROM↓0–9%. *60 months* (1 study, SNB): Muscle strength↓11%; pain 9%; LE 7%; axillary RT OR = 2.4; sleep disturbance 9%
**Tsai et al. 2009**	SR	ALND/SNB/RT/ALND vs. SNB n = 8262/Objective measurements n = 23964	98 studies: 10 RCT’s, 83 CCT: 40 prospective, 43 retrospective, 5 CSS/ALND vs. no ALND/13 studies/Radical mastectomy vs. other mastectomy 8 studies/1950–2008/% FU missing/1–360 months	Tape measurement, BIS, water displacement, self-report	**ALND vs. SNB:** LE RR = 3.07; **ALND vs. no ALND:** LE RR = 3.47; **Radical mastectomy vs. other mastectomy:** LE RR = 3.28; **RT axilla vs. RT no axilla:** LE RR = 2.97
**Lee et al. 2008**	SR	Surgery/RT not axilla/n = 5154/LE risk n = 2416/ROM↓ risk n = 476	25 studies: RCT 8; CCT24: prospective 17, retrospective 7/1966–2007/% FU missing/7 wks-203 months	ROM, VAS, tape measurement, water displacement, LENT-SOMA, EORTC-QLQ	**ALND vs. SNB**: ROM↓ 1%–67%; most problems 7–12 months post-surgery; muscle strength↓ 9%–28%; OR = 4.61; pain 9%–68%; OR = 3.03; LE 0%–34%; OR = 11.67; RT not axilla OR = 1.46; Shoulder complaints: OR = 9.8
**Ashikaga et al. 2010**	RCT	Stage not described/SNB + ALND vs. SNB (+ ALND in case of positive nodes)/RT/CT/n = 5611	36 months	Abduction ROM, water displacement	**ALND vs. SNB**: *2*–*3 weeks:* ROM: abduction↓: 56% vs. 21%. *6 months:*ROM abduction↓: 9% vs. 6%; ALND OR = 1.56; RT axilla OR = 2.48, CT OR = 0.73; LE: 13% vs. 9%. *12 months:*LE: 13% vs. 9%. *36 months:*LE: 14% vs. 8%; ↓ age (+/−50 years) OR = 1.41, dominant affected arm OR = 1.77, RT axilla OR = 3.47
**Andersen et al. 2012**	CCT	Stage not described/Surgery/RT/CT: CEF vs. CE+T/HT/n = 2893	35/24 months	NPRS, Sensory disturbances in hands and feet	Pain overall 53%; activities: 34% gave up. **CEF vs. CE+T:** Sensory disturbances in both hands: 15% vs. 23%; OR = 1.56. Sensory disturbances in both feet: 18% vs. 32%; OR = 2.00; in younger patients OR = 0.45; ↑ risk of giving up activities OR = 1.59
**Miller et al. 2012**	CCT	Stage not described/ALND vs. SNB/Mastectomy/n = 117	29 (3–64) months	Water displacement; perometer; LEFT-BC Questionnaire	**ALND vs. SNB:** LE: 3 vs. 0%; ALND: ↑ subjective symptoms; ↑Mean weight-adjusted water displacement change
**Ozcinar et al. 2012**	CCT	Stage I–II, cT1,2 N0/SNB vs. ALND/RT vs.. RT axilla vs. RT regional LN/n = 221	(99%); 64 (24–82) months	Tape measurement 10 cm above and below elbow	Lymphedema: *9*–*12 months:* 25%. *64 months:* 7% (↓by treatment LE)
**Taira et al. 2011**	CCT	ALND level I–III/Mastectomy vs. lumpectomy + RT/n = 196	FU 97% at 1 months; 96% at 6 months; 95% at 12 months; 80% at 24 months	FACT-G/FACT-B	**Mastectomy vs. lumpectomy + RT:** *1 month (severe):* ROM↓ 68 (15)% vs. 73 (14)%; muscle strength↓ 67 (10)% vs. 72 (18)%; pain 75 (18)% vs. 82 (20)%; lymphedema 27 (1)% vs. 41 (7)%; upper arm activities: Lifting↓ 83 (25)% vs. 88 (20)%; household chores ↓ 61 (4)% vs. 64 (13)%; self-care↓ 56 (4)% vs. 63 (9)%; physical activities↓ 73 (19)% vs. 76 (19)%. *1 year (severe):*ROM↓ 32 (4)% vs. 40 (7)%; muscle strength↓ 48 (7)% vs. 51 (5)%; pain 60 (12)% vs. 63 (7)%; lymphedema 26 (3)% vs. 48 (11)%; upper arm activities: Lifting↓ 34 (2)% vs. 39 (3)%; household chores↓ 28 (4) vs. 33 (1)%; self-care↓ 16 (0)% vs. 12 (1)%; physical activities↓ 41 (4)% vs. 39 (4)%. *2 years (severe):*ROM 23 (0)% vs. 30 (4)%; muscle strength↓ 39 (5)% vs. 56 (7)%; pain 42 (8)% vs. 56 (5)%; lymphedema 33 (10)% vs. 52 (15)%; upper arm activities: Lifting↓ 20 (1)% vs. 39 (4); household chores↓ 18 (1)% vs. 21 (3)%; self-care↓ 10 (0)% vs. 14 (4)%; physical activities: 34 (7)% vs. 31 (5)%
**Wernicke et al. 2011**	CCT	stage I–II/ALND vs. SNB/n = 265	119 months	ROM, tape measurement	**ALND vs. SNB:** ROM↓ ; Lymphedema 35% vs. 5%
**Land et al. 2010**	CCT	Node negative invasive BC/ALND vs. SNB/Mastectomy vs. lumpectomy/n = 747	36 months	Questionnaire adapted from DASH	ALND vs. SNB: Upper arm activities↓. *6 and 12 months:* ALND group: ↑ arm use avoidance. **Mastectomy vs. lumpectomy (+ ALND):** Lumpectomy: ↑ problems with shoulder/arm function, conducting social and work activities
**Yen et al. 2009**	CCT	Stage I–IV/ALND vs. SNB/Mastectomy vs. lumpectomy/RT/CT/HT/n = 1338	48 months	Telephone interviews: arm functioning related to LE, pain, or tenderness in the arm or hand on the side of surgery	Lymphedema 14% (self-report). ↑ LN removed: 6–10 nodes OR = 4.68; 11–15 nodes OR = 5.61; >16 nodes OR = 10.50
**Bevilacqua et al. 2012**	CoS	Stage II–IIIa/ALND level I–III/n = 1243	(84%); 60 months	Tape measurement	Lymphedema 30% at 60 months; curve ↓ increasing after 36 months. Nomogram <6 months: age, BMI, level of ALND; nomogram >6 months: age, BMI, level of ALND, seroma, early LE
**Levy et al. 2012**	CoS	Stages 0-III/ALND/SNB/-/Mastectomy/lumpectomy/Breast reconstruction/n = 115	>12 months	ROM, MRC-scale, NPRS, perometer, ULDQ, PAQ, BMI	*1 month:* ROM flexion/abduction↓ 60%; external rotation ↓ 25%. ROM↓: ALND, ↑ LN removed, mastectomy, stage II, hand dominant side, cording, seroma, BMI ≥25. ROM↑: ↑ level of PA. *12+ months:*Flexion/abduction 11/10%; external rotation ↓ 5%; muscle strength↓: 47%; pain 49% (11% moderate); fatigue 43%. ROM↓: positive LN, mastectomy (flexion), older age (>65 yrs), BMI ≥25. Heavy household chores ↓: feeling stiff OR = 4.60; feeling week OR = 9.67; pain OR = 6.16; LE OR = 4.16; fatigue OR = 9.33; lifting a gallon↓: feeling week: OR = 6.34; pain: OR = 4.58
**Mieog et al. 2012**	CoS	Stage I–III/Tamoxifen vs. exemestane/n = 4724	91 months	CTCAEv1 for CTS and MSD	CTS 2%; MSD 43%. **Exemestane vs. Tam:** OR = 9.90 for CTS. Independent risk factors: HT, history of musculoskeletal symptoms, arthralgia, myalgia, osteoarthritis
**Schmitz et al. 2012**	CoS	Stages I–III+/ALND vs. SNB vs. –/Mastectomy vs. lumpectomy/RT/CT/HT/n = 287	(70.7%); 72 months	tape measurement, BIS, DASH, FACT-B+4	Adverse effects: *6 months:*≥1: 90%; 2–4: 72%; >4: 16%; *12 months:* ≥1: 69%; 2–4: 46%; *18 months:* ≥1: 66%; 2–4: 34%; *72 months:*≥1: 62%; 2–4: 27%
**Kanematsu et al. 2011**	CoS	Stage 0-IV (1 x IV)/Aromatase inhibitors/CT/n = 391	40 (9–120) months	CTCAEv4	Age <55 vs. 55–65 vs. >65 years: Arthralgia 46% vs. 37% vs. 28%; pain frequency↑: ↓ age at menarche; pain frequency↓: time since last menstrual period >10 years; HT/CT/disease stage ns
**Ridner et al. 2011**	CoS	Stages I–IV/ALND/SNB/RT/n = 138	30 months	Perometer, Weight, LBCQ	Lymphedema 20% ; BMI ≥30 OR = 3.59; adjusted for ALND as risk factor OR = 4.12; 80% of LE patients heaviness
**Rief et al. 2011**	CoS	Early stage BC/Mastectomy/lumpectomy/HT/n = 2160	48 months	Symptom Inventory, METs, RAND36, Life Orientation Scale—Revised, MOS,	Pain ↑: pain or depression at baseline, life events first 12 months post-operative, TAM at baseline. Pain ↓ : ↑exercise, ↑ years since diagnosis, ↑ education. Pain scores↑: stage II lumpectomy, and stage I mastectomy
**Devoogdt et al. 2010**	CoS	Stage 0-IV/ALND/SNB/n = 267	(88%); 24 months	FPACQ, MET-hours/week	Activities: MET’s per week: *Preoperative:* 269; *3 months:* 244; *6 months:* 246; *12 months:* 258. MET’s↓: ↑ in younger age, being employed, ductal carcinoma
**Chang & Kim 2010**	CoS	Stage not described/Free flap, Latissimus dorsi flap/n = 482	17 months	missing	Lymphedema 8% pre-existing; 4%↑ after reconstruction; LE↓: delayed autologous reconstruction
**Johnsson et al. 2010**	CoS	Early stage BC/ALND/SNB/RT breast/chest wall/regional LN/CT/n = 100	10 months	Return to work 25%/5 hours; Li-Sat11; GCQ	Return to work: *6 months:* 66%; *10 months:* 83%. *Return to work↓:*At 6 months: CT, >30 days of sick leave during the previous 12 months, ↓ satisfaction with current capacity in ADL; at 10 months: RT breast/chest wall/regional LN, ↓ satisfaction with work
**Kwan et al. 2010**	CoS	Stages I–IV/ALND/SNB/RT/CT/n = 997	21 (1–32) months	CTCAE v.3.0; ICD; lymphedema treatment; compression device	Lymphedema: *12 months:* 10%; *24 months:* 14%. Model 1: ICIDH: African American, ↑education, each LN removed 4.1%↑; Model 2: LE treatment: CT; Model 3: Durable medical equipment associated with BC related LE: being obese
**Norman et al. 2010**	CoS	Stage I–IV/ALND/SNB/RT/CT/n = 4551	(86%); 12–60 months	Face to face interview followed by telephone interview	Lymphedema 14%. CT HR = 3.16; Multi-agent CT with anthracycline HR = 3.76
**Yang et al. 2010**	CoS	ALND/SNB/Mastectomy/Lumpectomy/Adjuvant treatment/n = 183	12 months	MPS, Hawkins’ test, supraspinatus test, and Neer’s test, PMPS, AWS, tape measurement	**ALND vs. SNB vs. lumpectomy:** Lymphedema 18%; upper arm activities ↓. *At 3 months:*39% vs. 18% vs. 12%; a*t 6 months:*40% vs. 12% vs. not described %; a*t 12 months:* 44% vs. 19% vs. 18%. Rotator cuff disease 12 months associated with pectoralis tightness and LE at 3 months
**Sagen et al. 2009**	CoS	Stage I–III/ALND level I–II/n = 204	60 months	VAS, water displacement, EORTC-QLQ-C30, self-generated questionnaire	*At 6 months:*Pain during activities vs. at rest 56% vs. 60% ; lymphedema 7%; upper arm activities: function scores ↓ (from 30 points to 29 points). *At 60 months:*Pain during activities vs. at rest 36% vs. 30% ; lymphedema 13%; physical activity at leisure time at baseline and 6 months predictive for physical functioning at 5 years
**Paskett et al. 2007**	CoS	Stage I–III Surgery/reconstruction/RT/CT/HT/n = 622	(93%); 36 months	BMI, self-generated questionnaire, SF12, FACT-B	LE 54%; predictive: tamoxifen
**Lundstedt et al. 2012**	CSS	Stage not described/ALND vs. SNB/RT vs. RT SC/n = 814	36–96 months	CTCAE	**ALND + RT vs. ALND vs. SNB/no RT:** LE 22% vs. 15 vs. 5%. LE↑: RT SC
**Sheridan et al. 2012**	CSS	Stage not described/Surgery/RT/CT/HT/n = 111	64 months	S-LANSS, CPAQ, HADS	Pain VAS 32±26. *Pre-operative:* 18%; Risk of chronic pain↑ OR = 5. *Post-operative:* 36%; 23% intermittent pain; 32% exacerbation by exercise; ↑ chronic pain related to anxiousness, CT
**Dahl et al. 2011**	CSS	Stage II–III/Surgery/RT/n = 337	30 months	Self-generated questionnaire, EORTC-QLQ-C30-BR23, FQ, HADS, SF-36	Pain arm/shoulder 37%; sleep disturbance 30%; ↑disability pension, depression, anxiety. Sleep disturbance ↑: arm/shoulder pain OR = 2.46; LE OR = 2.34; ↓ ROM OR = 2.63
**Nesvold et al. 2011**	CSS	Stage II–III/Surgery/RT/n = 349	(56%); 83–113 months	ROM flexion/abduction, tape measurement, KAPS, EORTC-QLQ-BR23, IOC, SF36	ROM ↓ 33%; pain sign. related to arm-shoulder problems; lymphedema 17%; upper arm activities ↓ 31%
**Shamley et al. 2009**	CSS	Stage not described/ALND vs. SNB/Mastectomy vs. lumpectomy/RT/CT/n = 152	6–72 months	Polhemus Fastrak™, SPADI	Pain: *0–24 months* 26%; *24–48 months* 43%; *48–72 months* 32%. Upper arm activities: *0–24 months* 26%; *24–48 months* 43%; *48–72 months* 32%. **Affected side vs. unaffected side:** All scapulothoracic movements sign. altered: Right scapulothoracic lateral rotation differences associated with downward movement; left scapulothoracic dysfunction (↑ protraction, ↑ posterior tilt, ↓ lateral rotation): CT. Pain and disability associated with scapulothoracic dysfunction; scapulothoracic movements: ↑ difference when left side affected
**Park et al. 2008**	CSS	Stage I–III/Mastectomy/RT/CT/n = 450	12–24 months	Tape measurement	Lymphedema 25%; disease stage (OR = 2.58 for stage II; OR = 2.84 for stage III); modified radical mastectomy OR = 7.48; ALND OR = 6.61; axillary RT OR = 6.73; CT; overweight OR = 2.01; non exercise vs. exercise OR = 1.24; not receiving pre-treatment education OR = 2.26; ↓ preventive self-care activities
**Ververs et al. 2001**	CSS	Stage not described/ALND/n = 400	3–60 months	Tape measurement, self-generated questionnaire	Muscle strength ↓ in 28%. Pain: comorbidity OR = 3.38. Lymphedema: Objective >2 cm 71%; severe LE 9%; RT SC/axilla OR = 3.57; comorbidity OR = 3.08. Shoulder, neck or back complaints: comorbidity OR = 2.72. Activities: 25–35% daily activities↓, lifting objects↓; 14% problems with transportation; 37% gave up hobbies or sports
**Avraham et al. 2010**	CCS	SNB +/− ALND/Mastectomy/Tissue expander/n = 316	60 months	LBCQ, tape measurement, BMI	**Reconstruction vs.. no reconstruction**: LE: 5% vs. 18% (severe <1% vs.. 4%); (overall 11% objective; 16% subjective). LE↑: Chest wall RT
**Mak et al. 2008**	CCS	ALND/n = 202/230	42±12/43±14 months	Tape measurement, validated questionnaire	LE↑: infection: OR = 3.80; ↑ age at surgery OR = 1.06 for each year. Moderate-severe LE: ALND dominant side, medical procedures on hand/arm, ↓ air travel, institution of surgery

Study design: CCT, clinical controlled trial; Cos, cohort study; CSS, cross sectional study; pts, patients; RCT, randomized controlled trial; SR, systematic review.

Intervention: ALND, axillary lymph node dissection; art, article; CE, cyclophosphamide, epirubicin; CEF, cyclophosphamide, epirubicin and fluorouracil; CT, chemotherapy; FU, follow up; Gy, Grey; HT, hormonal therapy; IMB, internal mammarial boost; IM-MS, internal mammary and medial supraclavicular lymph node chain; IORT, intra operative radiotherapy; LRRT, locoregional radiotherapy corresponding to periclavicular, axillary level 3, and for right-side breast cancers, the internal mammary nodes; LN, lymph node; M, metastasis; N, nodal status; PAB, posterior axillary boost; RT, radiotherapy; SC, supra scapular; SNB, sentinel node biopsy; T, docetaxel; T, tumor; TAM, tamoxifen; vs., versus; wks, weeks; ZOL, Zoledronic Acid.

Measurement instruments: BIS, bio impedance spectroscopy; BMI, body mass index; BSI, Brief Symptom Inventory; CPAQ, Chronic Pain Acceptance Questionnaire; CES-D, center for epidemiologic studies – depression scale; CTCAE, Common Terminology Criteria for Adverse Events ; DASH, disabilities of arm, shoulder and hand; EORTC-QLQ-C30-BR23, European organization for research and treatment of cancer – quality of life questionnaire- breast; FACT-G-B, functional assessment of cancer therapy – general – breast; FLIC, Functional living index – cancer; FQ, fatigue questionnaire; FPACQ, Flemish Physical Activity Computerized Questionnaire; GCQ, general coping questionnaire; HADS, hospital anxiety and depression scale; ICD, international classification of diseases; IOC, impact of cancer scale; KAPS, Kwan’s arm problem scale; LANSS, Leeds Assessment of Neuropathic Symptoms and Signs; LBCQ, lymphedema breast cancer questionnaire; LEFT-BC, Lymphedema Evaluation Following Treatment for Breast Cancer; LENT-SOMA, late effects normal tissue – subjective objective management analytic; Li-Sat, life satisfaction; MASS, measure of arm symptoms survey; MET, metabolic equivalent ; MOS, medical outcomes study; MPQ, McGill pain questionnaire; MRC-scale, medical research council scale; MSPQ, Modified Somatic Perception Questionnaire; NPRS, numeric pain rating scale; PAISSR, Psychological Adjustment to Illness Scale-Self-Report; PAQ, physical activity questionnaire; PSI-B, Problem solving inventory-brief; ROM, range of motion; SF-36, short form-36; SPADI, shoulder pain and disability index; ULDQ, upper limb disability questionnaire; v, version; VAS, visual analogue scale; WHR, Waist-Hip ratio.

Outcomes: ADL, activities in daily living; AWS, axillary web syndrome; CHF, cardiac heart failure; CTS, carpal tunnel syndrome; HR, Hazard Ratio; LE, lymphedema; LVEF, left ventricular ejection fraction; ns, non-significant; OR, odds ratio; MPS, myofascial pain syndrome; MSD, musculoskeletal disorders; PA, physical activity; PMPS, Post Mastectomy Pain Syndrome; RR, relative risk; sign, significant; *, data extracted from included studies.

### Quality assessment

We evaluated the methodological quality of the included studies to test generalizability and possible bias. Studies were rated using the Oxford Centre for Evidence-Based Medicine, 2011 appraisal sheets and levels of evidence (see table 3) [9]. Two authors (JH + CB) independently scored each item of the appropriate scoring sheet. Disagreements were discussed together or if appropriate in the research group. If the item was well described and its quality was good, a plus (+) was assigned, plus-minus (±) was assigned if the item was incompletely described, and minus (–) was used if the item was not clearly described or not described at all. Five items were used to score systematic reviews leading to a maximum score of 100% (see table 4 and 5). Only systematic reviews including meta-analysis could achieve a full score of 100%. For cohort studies, six items were scored. Since the type of surgical treatment may influence health outcomes, articles describing radiotherapy treatment not taking into account the type of surgical treatment were given no score to the item “Subgroups with different prognosis identified”. A full score was assigned to studies assessing the outcome “lymphedema” with measurements of the full arm, using tape measurements to calculate volume, water volumetry, perometry or bio-impedance spectroscopy (BIS). When other methods of multiple tape measurement were used, plus-minus was assigned to “validated outcome” criterion. If the Common Terminology Criteria for Adverse Events (CTCAE) was used as a measurement instrument for lymphedema no score was given, because only one location was measured. Questionnaires on lymphedema were given plus-minus, as these questionnaires led to a higher incidence percentage in relation to volumetric measurements. [10] In selecting studies with a quality score of >50% we aimed at reducing the risk of bias of the included studies resulting in more robust conclusions of our review.

**Table 3:** Oxford Centre for Evidence-Based Medicine, 2011 Levels of Evidence for common harms (Treatment harms).

Level 1	Systematic review of randomized trials, systematic review of nested case-control studies, *n*-of-1 trial with the patient you are raising the question about, or observational study with dramatic effect
Level 2	Individual randomized trial or (exceptionally) observational study with dramatic effect
Level 3	Non randomized controlled cohort/follow-up study provided there are sufficient numbers to rule out a common harm
Level 4	Case-series, case-control studies or historically controlled studies
Level 5	Mechanism-based reasoning

**Table 4:** Quality test of methodology of the included systematic reviews based on the critical appraisal sheets of the Centre of Evidence Based Medicine.

First author/year of publication	Search strategy	Inclusion criteria selection	Quality of the studies	Results homogeneous	Presentation of results	Rating
Hickey et al. 2013^37^	+	+	+	+	+	100%
Moja et al. 2012^14^	+	+	+	+/−	+	90%
Zhou et al. 2011^11^	+/−	+	+/−	+	+	80%
Liu et al. 2009^12^	+/−	+	+	+/−	−	60%
Tsai et al. 2009^1^	+/−	+	+/−	+	+	80%
Lee et al. 2008^15^	+	+	+	+/−	+	90%
Levangie et al. 2008^13^	+/−	+	+	+	−	70%

**Table 5:** Quality test of methodology of the included studies based on the critical appraisal sheets of the Centre of Evidence Based Medicine.

First author/year of publication	Study design	Inclusion in common point in the course of disease	Follow up sufficiently long and complete;Number of patients included-analyzed	Outcome criteria objective or based on “subjective judgement”	Subgroups with different prognosis, adjustment for prognostic factors	Results over time	CI stated and narrow	Rating
Ashikaga et al. 2010^35^	RCT	+	+	+	+	+	+	100%
Andersen et al. 2012^39^	CCT	−	+	+	+	−	+	67%
Miller et al. 2012^44^	CCT	+	−	+	+	−	+	67%
Ozcinar et al. 2012^18^	CCT	+	+	+/−	+	−	−	58%
Taira et al. 2011^28^	CCT	+	+	+	−	+/−	−	58%
Wernicke et al. 2011^19^	CCT	+	+	+/−	+	−	+	75%
Land et al. 2010^3^	CCT	+	+	+/−	+	+	+	92%
Yen et al. 2009^22^	CCT	+	+	−	+	+	+	83%
Bevilacqua et al. 2012^33^	CoS	+	+	+	−	+	+	83%
Levy et al. 2012^29^	CoS	+	+	+	+	+	+/−	92%
Mieog at al. 2012^24^	CoS	+	+	−	+	+/−	+	75%
Schmitz et al. 2012^4^	CoS	+	+	+	+	+	−	83%
Kanematsu et al. 2011^23^	CoS	+	−	−	+/−	+	+	58%
Ridner et al. 2011^45^	CoS	+	+	+	+	+	+	100%
Rief et al. 2011^30^	CoS	+	+	+	+/−	+/−	−	67%
Devoogdt et al. 2010^31^	CoS	+	+	+	+	+	+/−	92%
Chang & Kim 2010^25^	CoS	+	+	+	+	+	−	83%
Johnsson et al. 2010^42^	CoS	+	−	+	+	+/−	+	75%
Kwan et al. 2010^21^	CoS	+/−	−	+/−	+	+/−	+	58%
Norman et al. 2010^26^	CoS	+	+	+/−	+	+/−	+	83%
Yang et al. 2010^46^	CoS	+	+	+	+	+	+	100%
Sagen et al. 2009^40^	CoS	+	−	+	+	+	+	83%
Paskett et al. 2007^16^	CoS	+	+	−	+	+	+	83%
Lundstedt et al. 2012^27^	CSS	−	+	−	+	+/−	+	58%
Sheridan et al. 2012^41^	CSS	−	−	+	+	+/−	+	58%
Dahl et al. 2011^36^	CSS	+	+	+	+	−	+	83%
Nesvold et al. 2011^32^	CSS	−	+	+	+	+/−	−	58%
Shamley et al. 2009^38^	CSS	−	−	+	+	+	+	67%
Park et al. 2008^20^	CSS	+	+	+/−	+	+/−	−	67%
Ververs 2001^17^	CSS	−	−	+/−	+	+	+	58%
Avraham 2010^43^	CCS	−	+	+	+	−	+	67%
Mak et al. 2008^34^	CCS	−	+	+	+	−	+	67%

CCS-Case-Control Study; CCT, Clinical Controlled Trial ; CI, confidential interval; CoS, cohort study; CSS, Cross Sectional Study; RCT, Randomized Controlled Trial.

### Synthesis

First, we described detailed characteristics and the main findings of the included systematic reviews, RCTs, and cohort studies, as reported by the authors of the included studies. Second, we assessed adverse effects per impairment and activity limitations for each medical intervention and combination of medical interventions. Adverse effects were assessed for short-term impact (≤12 months follow-up) and long-term impact (>12 months follow-up). If a study did not identify which part of the treatment caused the adverse effects, the study was excluded from the analysis of outcome measures. Third, we assigned a level of evidence for each of the adverse effects related to the common harms of the medical intervention. [9] We anticipated on using a quantitative assessment in a meta-analysis, but due to the heterogeneity of outcome measures, adverse effects, and (combinations of) medical treatment we were unable to pool data from separate studies.

## Results

**Figure 1:**
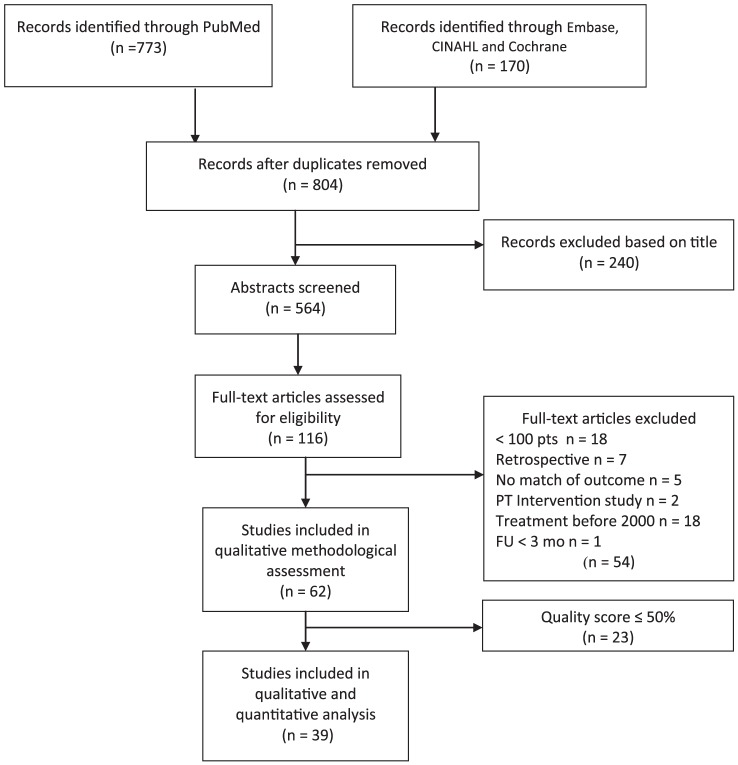
Flow diagram literature search adverse effects of breast cancer treatment.

We identified 804 unique articles, of which 116 were eligible for full-text assessment (see figure 1 for a flow diagram). Of these, 54 studies were excluded because they did not meet the inclusion criteria. Another 23 studies were excluded because they had already been included in one or more systematic reviews(15) or had a quality rating ≤50% (8). Finally, 39 articles were included. In the syntheses 13 articles could not be included because adverse effects were not analyzed separately for each treatment modality.

### Methodological quality of the included studies

The methodological quality of the included studies ranged from 60% to 90% for the systematic reviews (see table 4), and from 58% to 100% for prognostic studies and RCTs (see table 5). In four systematic reviews, the search strategy was limited to one database only. [1], [11]–[13] Results in four systematic reviews were not pooled due to the heterogeneity of the data. [11], [12], [14], [15] The majority of the cohort studies presented validated outcome measures, while seven of the 32 studies described outcome by a self-generated and self-validated questionnaire [3], [16], [17] or performed incomplete measurements. [18]–[21] In six studies, a description of the outcome was incomplete. [22]–[27].

### Adverse effects

Table 2 presents a detailed overview of the results of the included studies. Six systematic reviews and 29 cohort studies presented analyses regarding the origin of the adverse effects. Some studies analyzed the relationship of the adverse effects in relation to comorbidity, age or BMI.

In most studies, different subgroups were identified based on surgical treatment. Four studies [17], [28]–[30] focused only on patients that underwent ALND. One systematic review [1] and one cross-sectional study [27] focused on the adverse effects of radiotherapy. The adverse effects of aromatase inhibitors focused on musculoskeletal pain. [11], [22], [31] Zhou et al. described aromatase inhibitors in combination with zoledronic acids and pain. [11].

Synthesis per outcome measure is summarized and presented in table 6, including levels of evidence.

**Table 6:** Adverse treatment effects in relation to impairments in upper extremities and thorax.

	≤12 months post-surgery	>12 months post-surgery		Level of evidence
Medical intervention	%/p value/OR	OR/RR/HR	%/p-value	
Reduction in ROM				
ALND	1%–67%^15^		p = 0.0001^19^	level 2
SNB	*At 12 months:*6%–31%%^12^		*At 24 months:*0%–9%^12^	level 3
SNB + ALND vs. SNB	*At 12 months:* 24% vs. 24%/9% vs. 3%^13*^; a*t 6 months:* 56% vs. 21%; a*t 12 months:* 9% vs. 6%. OR = 1.56^32^	OR = 1.02/2.65/9.0^13*^	*At 18 months:* 8% vs. 4%; a*t* >*20 months:* 20% vs. 0%; a*t median 30 months* 11% vs. 4%^13*^	level 2
Mastectomy vs. lumpectomy		OR = 5.67 (CI = 1.03–31.16)^15^		level 1
ALND level I–III + mastectomy vs. ALND level I–III + lumpectomy + RT	*At 1 month:*68% vs. 73%; a*t 12 months:*32% vs. 40%^28^		*At 24 months:*23% vs. 30%^28^	level 3
RT chest wall vs. no RT		OR = 2.07/6.60/12.30^13*^; RR = 4.6^13^; OR = 2.48^32^	34% vs. 20%/38% vs. 4%/52% vs. 15%^13*^	level 2
RT axilla vs. no RT		RR = 2.6 (CI = 1.42–4.03)^1^; OR = 1.67 (CI = 0.98–2.86)^15^; OR = 2.48^35^		level 1
RT axilla + chest wall vs. RT chest wall		OR = 2.64/3.37^13*^	20% vs. 4%/Flexion 39% vs. 4%; 24% vs. 5%/Abduction 49% vs. 8%; 35% vs. 7%/External rotation 45% vs. 14%; 41% vs. 13%^13*^	level 2
CT vs. no CT		OR = 0.73, p = 0.003^32^		level 3
Reduction in muscle strength				
ALND		OR = 3.03 (CI = 1.25–7.32)^15^	28%^20^	level 1
SNB	17–19%^12^		*At 24 months:* 11%^12^	level 2
SNB + ALND vs. SNB	36% vs. 8%^13^	OR = 8.82^13^	48% vs. 16% ^13^	level 2
ALND + Lumpectomy	9%–28%^15^		OR = 4.61	level 1
ALND level I–III + mastectomy vs. ALND level I–III + lumpectomy + RT	*At 1 month:*67% vs. 72%; a*t 12 months:*48% vs. 51%^28^		*At 24 months:* 39% vs. 56%^28^	level 3
RT chest wall vs. no RT		OR = 1.70/3.37/6.83^13*^	14% vs. 2%^13^	level 2
RT axilla + chest vs. RT chest		RR = 1.7^13^	59% vs. 40%^13^	level 2
Concurrent RT + CT vs. sequential		OR = 2.09 (CI = 0.92–4.75)^36^		level 1
Pain				
ALND		OR = 4.61 (CI = 2.01–10.59)^15^	Shoulder pain 9%–68%^15^; Breast pain 15%–72%^14^; 53%^37^	level 1
SNB	8–36%^12^		*At 24 months:*8–21%; a*t 60 months:* SNB 9%^12^	level 2
SNB + ALND vs. SNB	*At 12 months:* 12% vs. 4%^13^	OR = 3.54 (1.88–6.66)^13^	*At 18 months:* 9% vs. 3%^13^	level 2
ALND level I–III + mastectomy vs. ALND level I–III +lumpectomy + RT	*At 1 month:* 75% vs. 82%; a*t 12 months:* 60% vs. 63%^28^		*At 24 months:* 42% vs. 56%^28^	level 3
RT vs. no RT		OR = 7.10^13^	*At 6*–*13 years:* weekly pain 26% vs. 4%^13^	level 2
RT before CT vs. RT after CT		Brachial neuropathy: OR = 3.14 (CI = 0.12–79.39)^36^		level 1
CT vs. no CT		OR = 3.00 (CI = 1.22–7.40)^40^		level 3
ZOL vs. no ZOL		Arthralgia: RR = 1.16 (CI = 1.096–1.232); Bone pain: RR = 1.26 (CI = 1.149–1.376)^11^		level 1
Delayed ZOL vs. upfront ZOL		Bone pain: RR = 1.28 (CI = 1.135–1.453)^11^		level 1
Exemestane vs. Tamoxifen		OR = 9.90 (CI = 3.52–27.82) for CTS^24^		level 3
Aromatase inhibitors; CT (with/without taxanes)			Age <55 vs. 55–65 vs. >65 yrs: Arthralgia 46% vs. 37% vs. 28%^23^; CTS 2%, MSD 43%^24^	level 3
Lymphedema				
ALND		RR = 3.47^1^; BMI >30: OR = 4.12 (CI = 1.58–10.72)^43^	0%–34%^15^/25%^20^/each LN removed 4.1% ↑^26^/HR = 2.61(CI = 1.77–3.84)^26^. *At 60 months:* 30%^33^/13%^39^	level 1
SNB	*At 6 month:* 3–10%; a*t 12 months:* 6–14%^12^		7%^12^	level 2
SNB + ALND vs. SNB	13% vs. 9%^35^/3% vs. 0%^44^	RR = 3.07 (no ALND 3.47)^1^/OR = 11.67 (CI = 1.45–93.65)^15^/OR = 6.61(CI = 1.64–26.57)^18^	35% vs. 5%^19^/14% vs. 8%^32^	level 1
Mastectomy		Radical mastectomy vs. other mastectomy RR = 3.28^1^; Modified radical mastectomy OR = 7.48 (CI = 2.38–23.85)^20^		level 1; level 3
ALND level I–III + mastectomy vs. ALND level I–III + lumpectomy + RT	*At 1 month:*27% vs. 41%; a*t 12 months:*26% vs. 48%^28^		*At 24 months:* 33% vs. 52%^28^	level 3
Reconstruction vs. no reconstruction			5% vs. 18%^42^	level 4
RT axilla vs. RT not axilla		RR = 2.97^1^/OR = 2.4^12^/OR = 3.57^17^		level 1
Concurrent vs. sequential RT after CT		OR = 2.02 (CI = 0.18– 22.61)^36^		level 1
RT before CT vs. RT after CT		OR = 2.11 (CI = 0.67–7.21)^35^		level 1
CT vs. no CT		HR = 1.46 (CI = 1.04–2.04)^26^		level 3
Reduction in level of activities in daily living				
ALND vs. SNB	↓arm use: p<0.001^3^	OR = 3.18/9.23^13*^		level 2
ALND + mastectomy vs. ALND + lumpectomy	shoulder/arm function, social and work activities: p = 0.001^3^			level 3
SNB + ALND vs. SNB vs. lumpectomy	*At 3 months:*39% vs. 18% vs. 12%; a*t 6 months:*40% vs. 12% vs. not described; a*t 12 months:* 44% vs. 19% vs. 18%^46^		Pain during activities vs. at rest 36% vs. 30%^40^/Daily activities↓, lifting objects↓ 25–35%; problems with transportation 14%; gave up hobbies or sports 37%^17^	level 2
ALND level I–III + mastectomy vs. ALND level I–III +lumpectomy + RT	*At 1 month:*Lifting↓ 83% vs. 88%; household chores 61% vs. 64%; self-care↓ 56% vs. 63%; physical activities↓ 73% vs. 76%. *At 12 months:*Lifting↓ 34% vs. 39%; household chores↓ 28 vs. 33%; self-care↓ 16% vs. 12%; physical activities↓ 41% vs. 39%^28^		*At 24 month:* Lifting↓ 20% vs. 39%; household chores↓ 18% vs. 21%; self-care↓ 10% vs. 14%; physical activities: 34% vs. 31%^28^	level 3
RT chest wall vs. no RT		OR = 1.32^13^	29 vs. 4%^13^	level 2
RT axilla + chest wall vs. RT chest wall		OR = 2.64/4.67^13*^		level 2
CE+T or CEF	34%^39^			level 3

Intervention: ALND, axillary lymph node dissection; CEF, cyclophosphamide, epirubicin and fluorouracil; CE+T, cyclophosphamide, epirubicin + docetaxel; CT, chemotherapy; HT, hormonal therapy; LN, lymph node; MRM, modified radical mastectomy; RM, radical mastectomy; RT, radiotherapy; SC, supraclavicular; SNB, sentinel node biopsy; vs., versus.

Outcomes: CTS, carpal tunnel syndrome; HR, hazard ratio; MSD, musculoskeletal disorder OR, odds ratio; RR, relative risk; ZOL, zoledronic acids; *, data extracted from included studies.

#### Reduction in range of motion (ROM)

Reduced ROM was described in four systematic reviews [1], [12], [13], [15] and six cohort studies. [19], [28], [32]–[35] General reduction in ROM was described [12], [15], [19], [28], [35] or specified for the shoulder in different directions: abduction, or flexion/abduction and external rotation. [32], [33].

Regarding ALND as a medical intervention, one systematic review reported a reduction in ROM in abduction and flexion ranging from 132–175°, which was reported in 1–67% of the patients. [15] Regarding SNB, a second systematic review described a reduction in ROM. [12] Percentages of patients with ROM reduction varied from 6%–31% after 12 months, and reduced to 0%–9% after 24 months. Regarding ALND (directly or after SNB) vs. SNB, change of ROM in the third systematic review was reported in 9%–56% vs. 3%–24% of the patients, or in a mean difference of 1°–20° within 12 months and 8%–20% vs. 0%–4% over 12 months. [13] Odds Ratios (ORs) in the included studies of this systematic review ranged from 1.02–9.0 for goniometric measurements. [13] One cohort study described a reduced ROM of 21% vs. 56% at 6 months and 6% vs. 9% at 12 months, with an OR of 1.56 at 12 months. [32] Another cohort study reported reduced ROM at six months and >12 months in a study population in which 71% underwent ALND. Reduction was present in 60% and 11% in flexion/abduction and 25% and 5% in external rotation [33]. ROM reduction was related to ALND, a greater number of lymph nodes removed, cording, seroma, mastectomy, stage II, hand dominance, BMI ≥25 and older age (>65 years).

Regarding mastectomy vs. lumpectomy, one systematic review presented an OR of 5.67 for mastectomy as a risk factor for reduced ROM. [15] In one cohort study, ROM reduction was present in 33% of the study population [34]. Mastectomy was indicated as risk factor. Regarding ALND and mastectomy vs. ALND, lumpectomy and radiotherapy reduced ROM was described at one, 12 and 24 months in overall percentages and percentages with severe reduction. Percentages reduced from 68% vs. 73% to 23% vs. 30%. [28]

Regarding radiotherapy vs. no radiotherapy, one systematic review presented ORs of 2.07–12.30, a relative risk (RR) of 4.6 and reduced ROM in 34%–52% vs. 4%–20% of the study population in the included studies. [13] One large cohort study presented an OR of 2.48 for radiotherapy as a risk factor for ROM reduction. [32] Regarding axillary radiotherapy vs. no axillary radiotherapy, the risk of decreased ROM was analyzed in two systematic reviews (RR 2.6; OR 1.67). [1], [15] A third systematic review reported changes in joint mobility in 14% vs. 2% of the patients in one included study; ORs in other included studies ranged from 1.70–6.83 for goniometric measurements. Regarding radiotherapy to the axilla and chest vs. radiotherapy to the chest, the same systematic review presented an RR of 1.7 in one included study and reduced ROM in 20%–49% vs. 4%–14% of the study population in other included studies. [13] Regarding chemotherapy vs. no chemotherapy, one large cohort study reported an OR of 0.73 of chemotherapy as a risk factor for ROM reduction. [32].

In synthesizing the results from the included studies, we found level 1 evidence for mastectomy and radiotherapy to the axilla as risk factors for reduced ROM in abduction, flexion and external rotation, and level 2 evidence for ALND and radiotherapy to the chest wall.

#### Reduction in muscle strength

Reduced muscle strength was reported in four systematic reviews [12], [13], [15], [36] and five cohort studies. [17], [18], [20], [33], [37].

Regarding ALND, one systematic review described reduced muscle strength (OR 3.03) [15]. One cohort study described reduced muscle strength in 28% of the study population [20]. Regarding SNB, a second systematic review reported reduced muscle strength in 17%–19% of the patients after sentinel node biopsy and 11% in the long-term. [12] This systematic review identified patients with young age (<50 years) as a risk factor for muscle strength impairment based on results of one large study comparing ALND vs. SNB. Regarding ALND (directly or after SNB) vs. SNB, a third systematic review reported weakness in 48% vs. 16% of the patients, with loss of abduction strength of 12–15 Nm, loss of grip strength of 12–41 Nm in the included studies and ORs ranging from 5.14–8.82 reported in the included studies. [13].

Regarding lumpectomy and ALND, one systematic review reported reduced muscle strength in9%–28% of the study population. [15] Regarding ALND and mastectomy vs. ALND, lumpectomy and RT reduced muscle strength was described at one, 12 and 24 months. [28] Percentages reduced from 67% vs. 72% to 39% vs. 56% reduced muscle strength. Reductions were larger in the first 12 months compared to later measurements (see table 6).

Regarding chest radiotherapy vs. no radiotherapy, the risk of reduced muscle strength was analyzed in one systematic review. [13] Extracted data from the included studies showed ORs from 1.70–6.83 for radiotherapy as a risk factor for reduced muscle strength and one included study reported reduced muscle strength in 14% vs. 2% of the patients. Regarding axillary radiotherapy vs. radiotherapy to the chest wall, the risk of reduced muscle strength was analyzed in the same systematic review. [13] One included study reported an RR of 1.7; another study showed 59% vs. 40% of the patients with reduced muscle strength. Regarding concurrent radiotherapy and chemotherapy vs. sequential radiotherapy and chemotherapy, a fourth systematic review described the risk of reduced muscle strength by concurrent treatment with an OR of 2.09. [36].

In synthesizing the results of the included studies, we found level 1 evidence for ALND, and concurrent radiotherapy and chemotherapy as risk factors for reduced muscle strength. We found level 2 evidence for SNB, radiotherapy to the chest wall and radiotherapy to the axilla and chest as risk factors for reduced muscle strength.

#### Pain

Pain was described in four systematic reviews [11], [12], [15], [36] and 10 cohort studies. [17], [22], [28], [31], [33], [35], [37]–[40].

Regarding ALND, one systematic review [15] and one cohort study [38] described pain 12 months post-operative. This systematic review described an OR of 4.61 and percentages of shoulder pain (9%–68%) and breast pain (15%–72%) in the individual studies. [35] The cohort study described pain in 53% of the population. [38] Regarding SNB, a second systematic review reported pain in 8%–36% of the patients within 12 months and 8%–21% at 24 months, analyzing young age (<50 years) as a predictive factor, described in one included study. [12] Regarding ALND (directly or after SNB) vs. SNB, a third systematic review reported pain during motion in one included study in 12% vs. 4% at 12 months and 9% vs. 3% at 19 months and an OR of 3.54 mentioned in another study. [13].

Regarding ALND and mastectomy vs. ALND, lumpectomy and radiotherapy pain was described at 1 month post-operatively, and at 12 and at 24 months. [28] Pain reduced from 75% vs. 82% to 42% vs. 56%. Regarding chest radiotherapy vs. no radiotherapy, one individual study in a systematic review reported at least weekly pain in 26% vs. 4% of patients (OR  =  7.10), 6 to 13 years post-operatively. [13] Regarding concurrent radiotherapy and chemotherapy vs. sequential radiotherapy and chemotherapy a fourth systematic review reported the risk of brachial neuropathy (OR 3.14). [36] Regarding chemotherapy vs. no chemotherapy, two cohort studies found chemotherapy to be a risk factor for pain, [38] with a reported OR of 3.00. [40].

Regarding the administration of zoledronic acids vs. no zoledronic acids, one systematic review reported the relative risk (RR) of arthralgia (RR 1.16) and bone pain (RR 1.26). [11] Regarding the upfront administration of zoledronic acids compared to delayed administration, the same systematic review described an increased risk of pain (RR 1.28). Regarding exemestane vs. tamoxifen, one cohort study described an increased risk of carpal tunnel syndrome (OR 9.90). [24] In this study, 43% of the patients had a musculoskeletal disorder and 2% carpal tunnel syndrome. Another cohort study described increased pain incidence by using tamoxifen at baseline and at younger age (< 55 years). [22].

In general, pre-operative pain was a risk factor for post-operative pain (OR 5.17) and prolonged pain. [24], [40] Pain was correlated with decreased muscle strength and range of motion, decreased job participation, reduced use of the affected arm in leisure activities and with lifting a gallon of milk or during heavy household chores. [33] At 6 months, pain during daily activities was less than at rest. [31], [41] In contrast, one study reported an exacerbation of pain by exercise. [40] Another study reported less pain during activities compared to rest at six months post-operative and more pain at 60 months. [39] Arm-shoulder pain led to sleep disturbances (OR 3.17). [35].

In conclusion, we found level 1 evidence for ALND, radiotherapy before chemotherapy, and the administration of zoledronic acids (more in case of delayed administration) as risk factors for pain. We found level 2 evidence for SNB and radiotherapy as risk factors for pain.

#### Lymphedema

Lymphedema was described in three systematic reviews [1], [12], [15] and 20 cohort studies. [4], [16]–[21], [23], [26]–[30], [32], [34], [39], [42]–[45] Eight studies reported subjective data based on a lymphedema questionnaire, [16], [23], [26], [28] CTCAE, [21], [27] telephone interview, [23], [26] or measured only 2 or 3 points of the arm. [18], [19].

Regarding ALND, two systematic reviews and five cohort studies described an increased risk of lymphedema. One systematic review described an RR of 3.47. [1] A second systematic review described percentages of pain in the included studies ranging from 0%–34%. [15] Percentages in the cohort studies varied from 13%–30%. [20], [29], [39] BMI ≥30 as a risk factor for lymphedema was described in one cohort study with an OR of 4.12 [44] and in another cohort study as an increase of 4.1% or HR of 2.61 for each lymph node removed. [26] Regarding SNB, a third systematic review described percentages ranging from 3%–14% in the first 12 months to 7% in the follow-up of 60 months. [12] Regarding ALND (directly or after SNB) vs. SNB, two systematic reviews and three cohort studies described lymphedema. One systematic review reported an RR of 3.07 (when compared to no axillary dissection 3.47), [1] while another systematic review reported an OR of 11.67. [15] In the cohort studies, percentages of patients with lymphedema varied from 3%–13% vs. 0%–9% in the first 12 months to 14%–35% vs. 5%–8% in longer follow up. [19], [32], [43].

Regarding mastectomy, lymphedema was described in one systematic review and one cohort study. The systematic review reported an RR of 3.28, [1] while the cohort study reported an OR of 7.48. [20] Regarding ALND and mastectomy vs. ALND, lumpectomy and radiotherapy lymphedema was described at one month post-operatively, and at 12 and at 24 months. [28] Percentages of patients with lymphedema increased from 27%–41% at one month to 33%–52% at 24 months post-operatively.

Regarding breast reconstruction vs. no reconstruction, one cohort study described lymphedema in 5% vs. 18% of the study population. [42].

Regarding radiotherapy to the chest and axilla vs. radiotherapy to the chest, two systematic reviews and one cohort study described lymphedema. One systematic review described an RR of 2.97, [1] the second an OR of 2.4. [12] The cohort study reported an OR of 3.57. [17] Regarding concurrent radiotherapy and chemotherapy vs. sequential radiotherapy and chemotherapy, one systematic review reported an OR of 2.02. [36] Regarding radiotherapy before chemotherapy vs. radiotherapy after chemotherapy, the same systematic review reported an OR of 2.11.

Regarding chemotherapy vs. no chemotherapy, one cohort study reported a Hazard Ratio (HR) of 1.46. [26] The risk of lymphedema in relation to chemotherapy was investigated in this cohort study in patients with ALND, comparing multi-agent chemotherapy with chemotherapy with anthracyclines. Regarding chemotherapy with radiotherapy vs. chemotherapy without radiotherapy, HRs in this study varied from 0.30–4.09 vs. 3.78–5.46.

The overall incidence of lymphedema increased over time, except in one study where lymphedema decreased because of decongestive lymphatic therapy. [18] One case control study described the risk of lymphedema due to infection in patients with ALND (OR 3.80). [30] BMI ≥30 as risk factor for lymphedema was described in one systematic review in patients with SNB as weak evidence, not providing data [12] and in two cohort studies (OR 3.59; adjusted for ALND OR = 4.1), [44] while an OR of 2.01 was found for BMI >25. [20] One study followed patients five years after ALND and provided nomograms that indicated a BMI >30 as a risk factor as well. [29] The influence of age on the development of lymphedema was described in one systematic review and four cohort studies, indicating young age (<50 years) [12], [16], [32] and age >65 years [30] as risk factors and increasing by age in another cohort study. [29].

One study reported that comorbidity led to a higher incidence of lymphedema. [17]

We found level 1 evidence for ALND, radical mastectomy, radiotherapy to the axilla, concurrent radiotherapy and chemotherapy, and radiotherapy before chemotherapy as risk factors for lymphedema.

#### Reduction in activities in daily living

Limitations in activities in daily living were described in two SRs [12], [13] and eight cohort studies. [3], [17], [28], [33], [38], [41], [45], [46].

Regarding ALND, one cohort study reported decreased degree of daily activities. [17] Regarding ALND vs. SNB one systematic review and one cohort study described an increased risk of problems in performing daily activities. [3], [13] ORs were calculated in two included studies in the systematic review (resp. 3.18 and 9.23). [13] Reported ORs for performing different tasks in one of the included studies in the systematic review varied from 2.13–2.34 when stratified by age, with age between 65 and 74 years at most risk and between 40 and 54 years at least risk compared to a non-breast cancer population. Decline in one or more tasks was described in another included study (34% vs. 50%, OR 0.8). One cohort study described the avoidance of normal arm use in cases of ALND compared to SNB (p <0.001). [3] Regarding ALND (directly or after SNB) vs. SNB vs. lumpectomy, one cohort study described a decline of activities in the first year post-operatively in 39%–44% of the patients after ALND, 18%–19% in case of SNB and 12%–19% in case of lumpectomy. [45] Regarding ALND and mastectomy vs. ALND and lumpectomy, one cohort study reported more problems in arm and shoulder function, conducting social activities and work in the lumpectomy group (p<0.001). [3] Regarding ALND and mastectomy vs. ALND, lumpectomy and radiotherapy, daily activities were described at 1 month post-operatively, at 12 and at 24 months in overall percentages and percentages with severe decline in daily activities. [28] Percentages reduced over time, with more problems in the lumpectomy group. Regarding chest wall radiotherapy vs. no radiotherapy, one systematic review reported a decline in daily activities with ORs in three individual studies (resp. 1.32, 8.0 and 10.67) and percentages of 29% vs. 4% in another included study. [13] Regarding radiotherapy to the axilla and chest wall vs. radiotherapy to the chest alone, the same systematic review reported an OR of 2.64 in one included study. Regarding chemotherapy with cyclophosphamide, epirubicin and docetaxel vs. chemotherapy with cyclophosphamide, epirubicin and fluoracil, one cohort study described a higher risk in giving up daily activities (OR 1.59). [38] Overall, 34% of the population in this study showed a decline in the level of daily activities.

Overall, one cross-sectional study described a decline in activities in 31% of the population. [34] One cohort study related radiotherapy to later starting remunerable work. [41] Activity level did not return to the pre-operative level within one year, [46] and at 10 months, 83% of the patients returned to work. [41] Young age as a predictive factor for a reduced number of metabolic equivalents was described in one cohort study. [46] Another cohort study described reduced use of the affected arm in leisure activities and with lifting a gallon of milk or during heavy household chores in relation to pain and feeling weak. [33].

Comorbidity was related to a decreased level of activities in daily living. [17].

We found level 2 evidence for ALND and radiotherapy, especially when the axilla was involved, as risk factors for decreasing the degree of daily activities.

## Discussion

In this systematic review, we showed that breast cancer treatment results in multiple impairments in the arm and shoulder. We analyzed adverse effects for different components of breast cancer treatment and related these to the integrated treatment of breast cancer. Previous systematic reviews, as well as a part of the cohort studies included in this study, merely focused on only a part of the medical treatment and/or outcome measurements, while others only looked at a general level, without distinction between components. By distinguishing between each treatment modality and outcome measurement, we are the first to analyze the risk of each component of breast cancer treatment. We showed that patients treated with ALND are at the highest risk of developing impairments of the arm and shoulder. Reduced ROM and muscle strength, pain, lymphedema and decreased degree of activities in daily living were reported most frequently in relation to ALND. Lumpectomy was related to a decline in the level of activities of daily living. Radiotherapy and hormonal therapy were the main risk factors for pain.

An integrated approach in assessing the adverse effects of distinct breast cancer treatment modalities on impairments in arm and shoulder function is of clinical importance. Recovery from adverse effects can be addressed in multidisciplinary treatment of patients; for example, physical therapy may be suitable for the recovery of ROM, muscle strength, lymphedema and daily activities. In general, we expect that awareness and timely referral are very relevant for patients with impairments interfering with daily activities in early recovery [47]. More attention should be paid to scapular coordination and muscle strength in the early post-operative phase, as these impairments were reported even up to six years post-operatively. [12], [13], [15], [37] We noticed that the included studies focused more on impairments in function than on activities of daily living or participation in remunerable work, hobbies and social activities. In future research, more awareness of these issues is warranted, as performing activities is an important outcome for quality of life. This will further build the body of knowledge for regaining full recovery of activities of patients with breast cancer in a multidisciplinary approach.

Unfortunately, due to the large variety in medical treatments and outcome measures, we could not perform a meta-analysis of our data. This emphasizes the importance of uniform description of treatment, analysis of outcomes, and use of uniform measurement instruments. Validated measurement instruments are important in assessing outcomes of treatments. We found a large variability of instruments, which made it difficult to compare studies and conduct a meta-analysis. This conclusion was also stated by authors of several included systematic reviews in our study [12], [13], [15]. International consensus regarding measurement instruments and the way of using them should be encouraged.

From our review it became clear that reduced ROM, pain and lymphedema are the most commonly described impairments. ROM decreased, especially in the first month post-operatively. As most systematic reviews presented data only for long-term follow-up after treatment, reductions in the first month were less noticed, but when described in cohort studies significance existed. After 12 months, percentages of patients with reduction in ROM and differences in ROM between the affected and unaffected shoulder were reduced but still existed. Wide variation of percentages shows the variability in defining ROM impairment and the way of measurement.

The incidence of lymphedema increased over time. One study reported a very high incidence of lymphedema after one month. [28] This may be due to real lymphedema or rather seroma or radiotherapy-induced breast infection. [48].

The study of Ozcinar et al. [18] showed that treatment of lymphedema decreased its severity. In general, the reported percentages of patients with lymphedema were higher when lymphedema was measured by a questionnaire. The Norman questionnaire appeared to be sensitive for detection, but not specific, [10] and may be used as an initial tool in detecting lymphedema. Volume is the most important outcome for lymphedema diagnosis and treatment evaluation; therefore, the questionnaire should be followed by tape measurement (calculated to volume) or water volumetry or perometry. Arm volume is also associated with Body Mass Index and body composition. Therefore we advocate to use percentage difference between arms (where A is the affected arm and U is the unaffected arm)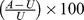 or to use the formula for relative volume change (RVC) to determine outcome over time. 
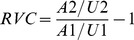



Activities in daily living and participation are important parameters for quality of life. Limitation in body functions and structures may be restrictive in performing activities and participating in social events. Only one systematic review [13] and six cohort studies [3], [17], [28], [38], [41], [46] described limitations in activities and only three cohort studies described problems in participation. As half of the patients with breast cancer were of working age, more attention should be paid to daily activities, work capacity, hobbies and sports.

Several limitations to our study should be noted. Our cut-off point with a quality score >50% is to some extent arbitrary and may have resulted in the exclusion of valuable data in our analysis. Main reasons for the low quality scores of excluded studies were issues with subgroup analysis, lack of outcome measures, poor presentation of results and lack of sufficient follow-up. Firstly, we analyzed which articles in our search were included in the systematic reviews. Four systematic reviews were excluded: based on treatment before 2000 or with low quality score. The review with low quality score was narrative and based on retrospective data. We therefore think the exclusion of these studies has avoided bias and contribute to the robustness of our conclusions. Based on the homogeneity of the results our choice seems to be justified. Another point is that, instead of relying on the review synthesis, it would have been a possibility to use existing reviews as sources to identify primary data, which would increase the value of the paper. We choose to follow the recommendations according the Oxford Centre of Evidence-Based Medicine. In this system systematic reviews are one of the factors in evidence classification. If it would have been possible to perform a meta-analysis the original data would have been extracted from the reviews. However, as described, this was not possible. We deemed additional analysis not to be of added value for the purpose of our paper. Therefore we used quality scores to test the credibility of the conclusions of the original authors and used these in the synthesis. Adverse effects of radiotherapy that may influence limitations in arm and shoulder function, such as fibrosis of the skin and sub cutis, were not included in our study. In addition, adverse effects of chemotherapy and target therapy on general cardiopulmonary capacity were not included. Other reported symptoms such as sleep disturbances, weight gain, cardiac function and sensory disturbances have not been reported, as have anxiety and depression, while these problems may influence the capacity of performing daily activities.

## Conclusions

Patients with breast cancer suffer from constraints in arm and shoulder in the first year post-operative and at long-term follow-up. Patients treated with ALND are most at risk for developing impairments of the arm and shoulder. Reduced ROM and muscle strength, pain, lymphedema and decreased degree of activities in daily living were reported most frequently in relation to ALND. Lumpectomy was related to a decline in the level of activities of daily living. Radiotherapy and hormonal therapy were the main risk factors for pain.

An integrated approach in addressing the adverse effects of distinct breast cancer treatment modalities on impairments in arm and shoulder function is of clinical relevance. Patients treated with ALND require special attention to detect and consequently address impairments in the arm and shoulder. Patients with pain should be monitored carefully, because pain limits the degree of daily activities.

## Supporting Information

Checklist S1 (DOC)
